# Determinants of exclusive utilisation of scheduled and unscheduled primary care visits: a cross-sectional study in Portugal

**DOI:** 10.1186/s12875-026-03174-z

**Published:** 2026-01-20

**Authors:** João Carneiro, Paulo Santos

**Affiliations:** 1 USF Cuidar, ULS Entre Douro e Vouga, Santa Maria da Feira, Portugal; 2 Faculty of Medicine, University of Porto, Porto, Portugal; 3 MEDCIDS, RISE-Health@RISE, Faculty of Medicine, University of Porto, Porto, Portugal

**Keywords:** Primary health care, Health services accessibility, Chronic disease, Patient scheduling, Cross-Sectional studies

## Abstract

**Background:**

Primary healthcare (PHC) is essential for delivering accessible, continuous, and comprehensive care. While scheduled and unscheduled visits are both integral to PHC, the exclusive use of one type has not been previously explored. Understanding the determinants of exclusive utilisation may inform service organisation and improve efficiency.

**Methods:**

This cross-sectional study included adults (≥ 18 years) who consulted a family physician in the Local Health Unit of Entre-Douro-e-Vouga, Portugal, during 2023. Patients who used both scheduled and unscheduled visits were excluded from the analysis. Data were extracted from anonymised electronic health records. Sociodemographic and clinical variables were analysed using multivariate logistic regression to identify factors associated with exclusive appointment type.

**Results:**

Among 28,213 patients, corresponding to 10.1% of the total, 68.0% used only scheduled visits and 32.0% only unscheduled visits. Scheduled visits were more frequent among women, local residents, employed individuals, and patients with diabetes, hypertension, obesity, cancer, or depression. Unscheduled visits were associated with men, students, and patients with asthma, dementia, anxiety, or cerebrovascular disease. Registration at a Family Health Unit (USF) was independently associated with higher odds of unscheduled visits (OR 0.54; 95% CI 0.50–0.59). Chronic conditions requiring structured follow-up were strong predictors of scheduled visits, while episodic or fluctuating conditions were linked to unscheduled utilisation.

**Conclusions:**

Exclusive utilisation patterns in PHC are shaped by sociodemographic and clinical factors. Scheduled visits reflect engagement with preventive care and chronic disease management, whereas unscheduled visits respond to acute or unpredictable needs. These findings highlight the need to tailor care pathways to patient profiles and ensure that organisational models—such as USFs—balance accessibility with continuity. Strengthening structured follow-up and improving responsiveness for episodic conditions may enhance equity and efficiency in primary care delivery.

## Introduction

Primary healthcare (PHC) is the cornerstone of population health, providing accessible, comprehensive, and continuous care. Sociodemographic characteristics, healthcare organisation, and patient preferences influence patterns of PHC utilisation. Increased demand for services and workforce shortages may compromise access to and efficiency of services [1]. The spatial distribution of health centres and physician availability also affects avoidable hospitalisations and continuity of care [2]. Scheduling models have been shown to impact patient satisfaction and service quality [3].

In Portugal, PHC is the first entry point into the National Health Service, with family physicians acting as both care providers and gatekeepers to specialist care. Since 2006, PHC has been organised into Family Health Units (USFs, from Portuguese *Unidades de Saúde Familiares*), with greater autonomy and a pay-for-performance scheme, and conventional health centres, with less flexibility [4].

Both provide scheduled and unscheduled consultations. Scheduled appointments are typically for routine, follow-up, or preventive care, whereas unscheduled visits respond to immediate health needs. Previous studies in Portugal examined appointment scheduling, travel time, and waiting periods [1, 5]. However, no research has explored the determinants of exclusive use of scheduled or unscheduled PHC visits. This study addresses this gap by characterising sociodemographic and clinical factors associated with exclusive utilisation of each type of visit.

## Methods

### Study design and setting

This observational cross-sectional study analysed adults (≥ 18 years) consulting a family physician in the Local Health Unit of *Entre-Douro-e-Vouga* (Aveiro district, Portugal) between January and December 2023. The unit serves over 274,000 residents across five municipalities, with 97% assigned to 194 family physicians in 24 USFs and eight conventional health centres.

### Participants

We included adults with at least one PHC consultation in 2023. We did not include children and adolescents under 18 because they are managed under pediatric programs, which encompass the entire population.

### Data collection

Anonymised data were extracted from electronic medical records, including sociodemographic variables (age, sex, residence, and employment status), as well as health conditions (hypertension, diabetes, overweight/obesity, smoking, alcohol misuse, cancer, cerebrovascular disease, cardiac disease, COPD, asthma, dementia, depression, and anxiety). The educational level was excluded due to the high rate of missing data (> 85%).

### Statistical analysis

Descriptive statistics summarised participant characteristics. Group comparisons utilised the Mann-Whitney U test (for continuous variables), the Chi-square test (for categorical variables), and logistic regression (for multinomial variables). Multivariate logistic regression, adjusted for age, sex, and number of visits, identified determinants of appointment type. Employment status was included as an independent explanatory factor rather than an adjustment variable, as it reflects organisational determinants of access rather than demographic confounders. Results are reported as odds ratios (ORs) with 95% confidence intervals (CIs). Significance was set at *p* < 0.05.

### Ethical considerations

The study was approved by the Ethics Committee of the Local Health Unit of *Entre-Douro-e-Vouga* (Process number 26/2024, of June 04, 2024) and conducted in accordance with the principles outlined in the Declaration of Helsinki and the Convention of Oviedo. The data were collected from an irreversibly anonymised database. The ethics committee waived the requirement for participants’ individual informed consent, as per Portuguese law (Law n.º 21/2014, of April 16, with the alterations introduced by Law n.º 49/2018, of August 14, and Law n.º 73/2015, of July 27).

## Results

Among 280,072 patients who attended at least one medical appointment in 2023, we included 28,213 patients (10.1%). From these, 19,184 (68.0%) had solely scheduled visits, and 9,029 (32.0%) had only unscheduled visits. The mean age was 50.0 years (± 18.6), and women represented 53.0% of the total sample (Table 1).

**Table 1:** Determinants of exclusive schedule and unscheduled appointments in primary healthcare in the local health unit of Entre-Douro-e-Vouga

Social Characteristics	Total *n* = 28,213	Scheduled *n* = 19,184	Unscheduled *n* = 9029	*p*-value
Women, n (%)	14,959 (53.0)	10,405 (54.2%)	4,554(50.4%)	< 0.001 *
Age (years), mean (SD)	50.02 (± 18.7)	50.9 (± 18.4)	48.1 (± 19.0)	< 0.001 **
Number of appointments, mean (SD)	2.57 (± 2.25)	2.65 (± 2.29)	2.40 (± 2.13)	< 0.001 **
Registered in a USF, n (%)	21,798 (77.3)	14,209 (77.9)	7,589 (85.5)	< 0.001 *
Local residents, n (%)	26,055 (92.4)	17,918 (93.4)	8,137 (90.1)	< 0.001 *
Work situation				< 0.001 *
Employed, n (%)	14,878 (52.7)	10,528 (59.6)	4,350 (55.3)	Ref
Student, n (%)	5,375 (19.1)	3,535 (20.0)	1,840 (23.4)	< 0.001 ***
Unemployed, n (%)	3,084 (10.9)	2,203 (12.5)	881 (11.2)	< 0.001 ****
Retired, n (%)	2,192 (7.8)	1,401 (7.9)	791 (10.1)	0.455 ***
Any disease, n (%)	3,326 (11.8)	2,280 (11.9)	1,046 (11.6)	0.466 *
CVD risk factor, n (%)	16,087 (57.0)	11,559 (60.3)	4,528 (50.1)	< 0.001 *
Arterial hypertension, n (%)	5,881 (24.3)	4,653 (24.3)	1,228 (13.6)	< 0.001 *
Diabetes, n (%)	1,988 (7.0%)	1,818 (9.5)	170 (1.9)	< 0.001 *
BMI > 25, n (%)	13,880 (49.2)	10,035 (52.3)	3,845 (42.6)	< 0.001 *
Smokers, n (%)	2,058 (7.3)	1,347 (7.0)	771 (7.9)	0.010 *
Alcohol abuse, n (%)	295 (1.0)	212 (1.1)	83 (0.9)	0.152 *
Cardiac disease, n (%)	1,199 (4.2)	853 (4.4)	346 (3.8)	0.017 *
Cerebrovascular disease, n (%)	497 (1,8)	330 (1.7)	167 (1.8)	0.441 *
Any cancer, n (%)	884 (3.1)	655 (3.4)	229 (2.5)	< 0.001 *
Asthma, n (%)	1,148 (4.1)	705 (3.7)	443 (4.9)	< 0.001 *
COPD, n (%)	373 (1,3)	265 (1.4)	108 (1.2)	0.204 *
Anxiety, n (%)	1,476 (5.2)	962 (5.0)	514 (5.7)	0.017 *
Depression, n (%)	2,109 (7.5)	1,489 (7.8)	620 (6.9)	0.008 *
Dementia, n (%)	28(0.1)	9 (0.0)	19 (0.2)	< 0.001 *

* chi-square test; ** Mann-Whitney U test; *** Logistic regression

SD: Standard deviation; USF: Family Health Units; CVD: Cardiovascular disease; BMI: Body mass index; COPD: Chronic Obstructive Pulmonary Disease

Scheduled visits were more common among women, older adults, local residents, and individuals who were employed or retired. Chronic conditions such as diabetes, hypertension, obesity, cancer, cardiovascular disease, and depression were significantly associated with scheduled visits.

Unscheduled visits were more frequent among men, students, and patients with asthma, dementia, anxiety, or smoking habits. Registration at one USF was linked to higher unscheduled utilisation.

In multivariate analysis, independent predictors of scheduled visits included female sex (OR 1.27; 95% CI 1.21–1.35), diabetes (OR 4.59; 95% CI 3.83–5.49), hypertension (OR 1.70; 95% CI 1.56–1.85), and obesity (OR 1.23; 95% CI 1.16–1.30). Conversely, asthma (OR 0.68; 95% CI 0.59–0.77), dementia (OR 0.28; 95% CI 0.10–0.74), anxiety (OR 0.70; 95% CI 0.62–0.79), and registration in a USF (OR 0.54; 95% CI 0.50–0.59) predicted unscheduled visits (Table 2).

**Table 2:** Multivariate analysis of factors associated with exclusive use of scheduled versus unscheduled visits

Factors	OR (95% CI)	*p*-value *
Female vs. Male	1.274 (1.205–1.348)	< 0.001
Age	1.000 (0.998–1.002)	0.962
Number of appointments (2023)	1.002 (0.989–1.016)	0.750
Registered at one USF	0.543 (0.503–0.588)	< 0.001
Work situation		< 0.001
Employed	< ref>	
Student	0.934 (0.842–1.037)	0.201
Unemployed	0.862 (0.800-0.928)	< 0.001
Retired	0.712 (0.639–0.792)	< 0.001
Diabetes	4.587(3.831–5.491)	< 0.001
Hypertension	1.697 (1.556–1.851)	< 0.0.001
Cerebrovascular disease	0.587 (0.474–0.726)	< 0.001
Asthma	0.675 (0.593–0.769)	< 0.001
BMI > 25	1.227 (1.158–1.301)	< 0.001
Dementia	0.275 (0.102–0.741)	0.011
Anxiety	0.702 (0.624–0.791)	< 0.001

Multivariate logistic regression of type of appointment selection adjusted for age, gender, and number of appointments (CI: confidence interval; OR: odds ratio; *p*-value was set at < 0.05); CVD = Cardiovascular Risk Factors; COPD = Chronic Obstructive Pulmonary Disease; BMI = Body Mass Index; USF = Family Health Units. CVD risk factors include hypertension, diabetes, BMI over 25 Kg/m^2^, tobacco and alcohol use. Any known disease included patients with the history of transient ischemic attack, stroke, stable angina, unstable angina, acute myocardial infarction, cardiac insufficiency, cancer, COPD or Asthma. Cardiac disease includes the history of stable angina, unstable angina, acute myocardial infarction or cardiac insufficiency. Cerebrovascular disease consists of a history of transient ischemic attack or stroke

## Discussion

Our results show that sociodemographic and health-related factors are closely associated with the exclusive use of scheduled or unscheduled primary care visits in Portugal. By focusing on patients who consistently used only one type of appointment during a year, this study provides a more apparent distinction between utilisation patterns, thereby avoiding the confounding effects of mixed users who alternate according to episodic needs or appointment availability. This methodological choice strengthens the interpretation of determinants that underlie consistent behaviours and informs targeted interventions.

### Sociodemographic factors

The choice between scheduled and unscheduled consultations reflects the complexity of patient behaviour in primary care. Female patients were more likely to schedule appointments, possibly reflecting their greater involvement in preventive programmes, such as cervical cancer screening and maternal health initiatives [6]. Women are also more likely to value continuity of care with the same practitioner [7]. In contrast, men tend to rely more on unscheduled consultations, which may reflect less engagement with preventive care and a greater likelihood of timeliness issues [8, 9]. Contrary to previous studies [6–10], age was not a significant determinant in our multivariate analysis. This divergence suggests that, in this context, multimorbidity, polypharmacy, and perhaps other factors, such as accessibility in older patients [11], may generate both scheduled follow-up and unexpected acute needs, making age alone a weaker predictor.

Employment status also influenced utilisation. Employed individuals sought more scheduled consultations, while retired and unemployed patients were less likely to use them, possibly due to reduced engagement with preventive care or difficulties navigating appointment systems. These findings underscore the importance of tailoring scheduling models and outreach strategies to diverse social contexts.

### Clinical conditions

Hypertension, diabetes, and being overweight were strongly associated with scheduled visits [12]. These conditions represent major cardiovascular risk factors [13] and require ongoing monitoring through structured, guideline-based follow-up [14–16]. Family physicians play a central role in preventing complications, with follow-up pathways often driven by protocol [17]. Our findings confirm that PHC in Portugal effectively integrates chronic disease management into scheduled primary healthcare services. Cancer and depression also increased the likelihood of scheduled consultations, underlining the relevance of both physical and mental health conditions in driving regular follow-up.

### Conditions linked to unscheduled use

Conversely, episodic or unpredictable conditions were linked to unscheduled utilisation. Asthma, dementia, anxiety, and stroke were more often managed through reactive care. This may reflect a mismatch between standardised scheduling systems and the fluctuating needs of these patients [18–22]. Asthma exacerbations and post-stroke complications, for example, often require urgent intervention, which routine appointments cannot fully address [18].

Patients with dementia were especially prone to unscheduled visits. Previous Portuguese studies have reported challenges in dementia care within PHC, including limited team training and fragmented implementation of the national dementia strategy [23]. These gaps may explain why patients and families rely on urgent consultations rather than scheduled follow-up. Similarly, individuals with anxiety disorders — highly prevalent and often under-recognised in PHC—showed greater reliance on unscheduled visits. This aligns with previous studies highlighting the high burden of mental health conditions on unplanned care [22, 24, 25].

### Organisational factors

Registration in Family Health Units (USFs) was independently associated with greater unscheduled use. Although USFs are generally more efficient and accessible [26], their flexibility and proximity may facilitate same-day or walk-in consultations [11]. This paradox suggests that increased accessibility may sometimes lead to reliance on unscheduled visits, raising questions about the continuity of care and the balance between responsiveness and proactive management.

### Strengths and limitations

The strengths of this study include the large sample size, population-based design, and robust statistical analysis. The focus on exclusive users provided clear behavioural profiles, rarely studied in the literature. That’s why we excluded the mixed users to avoid confounding effects. However, the cross-sectional design limits causal inference, and excluding mixed users, although methodologically justified, reduces generalisability, since most patients combine both types of visits. Future longitudinal studies should assess transitions between scheduled and unscheduled utilisation and their implications for care continuity.

### Implications for practice and policy

Our results have direct implications for the organisation of PHCs. First, scheduled care pathways for chronic disease management should be strengthened to maintain structured follow-up. Second, more flexible models are required for conditions characterised by unpredictable courses, such as asthma, dementia, and anxiety. Finally, policies should leverage the advantages of USFs while ensuring that accessibility does not undermine continuity, thus achieving a balance between preventive and responsive care.

## Conclusion

This study highlights the importance of understanding exclusive patterns of scheduled and unscheduled primary care utilisation, revealing how sociodemographic and clinical factors shape access to different types of care. The findings suggest that the current organisation of primary care in Portugal may be well-suited for patients with cardiovascular risk factors and those eligible for cancer screening, but less responsive to individuals with more unpredictable or episodic needs, such as those with asthma, stroke, dementia, or anxiety.

This is undoubtedly a potential gap in continuity and equity, underscoring the need for a more balanced approach in primary care delivery. This approach should maintain the strengths of preventive care while ensuring timely and flexible responses to acute and complex conditions. In Portugal, Primary Care physicians are specialists in General and Family Medicine, which attempts to align English-inspired general practice with American family medicine. Reconciling these two perspectives isn’t always easy, and one often ends up leaning more heavily on one side than the other. These results remind us of the need for thoughtful consideration and the development of organisational strategies that allow us to be a family doctor while remaining a general practitioner.

## Data Availability

The data supporting the findings of this study are available from the authors upon reasonable request and with a justified reason, by contacting the corresponding author.

## Abbreviations

BMI: Body Mass Index
CI: Confidence Interval
COPD: Chronic Obstructive Pulmonary Disease
CVD: Cardiovascular Disease
OR: odds ratio
PHC: Primary Health Care
SD: Standard Deviation
USF: Family Health Unit (from Portuguese: Unidade de Saúde Familiar)
